# Comprehensive Evaluation of Volatile and Nonvolatile Compounds in Oyster Cuts of Roasted Lamb at Different Processing Stages Using Traditional Nang Roasting

**DOI:** 10.3390/foods10071508

**Published:** 2021-06-29

**Authors:** Yujun Xu, Dequan Zhang, Huan Liu, Zhenyu Wang, Teng Hui, Jilu Sun

**Affiliations:** 1 College of Food Science and Technology, Hebei Agricultural University, Baoding 071000, China; xuyujun_96@163.com; 2 Institute of Food Science and Technology, Chinese Academy of Agricultural Sciences, Key Laboratory of Agro-Products Processing, Ministry of Agriculture and Rural Affairs, Beijing 100193, China; zhangdequan@caas.cn (D.Z.); liuhuan02@caas.cn (H.L.); wangzhenyu@caas.cn (Z.W.)

**Keywords:** flavor attributes, volatile compounds, free amino acids, 5′-nucleotides, Nang roasted lamb

## Abstract

Nang roasting is a traditional lamb processing method in Xinjiang (China) with a history of thousands of years. This study comprehensively evaluated the volatile and nonvolatile compounds of oyster cuts of roasted lamb at different processing stages of Nang roasting using gas chromatography mass spectrometry and amino acid automatic analyzer, respectively. Results indicated that aldehydes were the dominant profiles of volatile compounds, and hexanal, nonanal, octanal, (E)-2-nonenal, (E, E)-2,4-decadienal, (E, E)-2,4-nonadienal and 1-octen-3-ol were the key volatile compounds or aroma contributors to roasted oyster cuts. Isoamylol and 3-hydroxy-2-butanone could differentiate fresh and marinated oyster cuts from roasted ones; (E)-2-nonenal, (E, E)-2,4-decadienal, 1-octen-3-ol, hexanal, octanal, nonanal and (E, E)-2,4-nonadienal could differentiate Nang roasted oyster cuts of 60 min from those of 15, 30 and 45 min. Umami amino acids and sweet amino acids are the dominant profiles of nonvolatile compounds; glutamic acid, alanine and 5′-IMP were the key free amino acids or taste contributors to roasted oyster cuts. Glutamic acid, alanine and 5′-IMP could differentiate fresh and marinated oyster cuts from roasted samples. This work provided theoretical support for the control of flavor attributes of roasted lamb with traditional Nang roasting.

## 1. Introduction

Roasting can provide meat products with a unique flavor [1] and roasted meat is popular among almost all consumers worldwide. Nang roasted lamb leg is one of the traditional meat products with classical flavor attributes and a history of thousands of years, which is processed by special thermal treatment named Nang roasting from the Xinjiang Uyghur people. Briefly, during Nang roasting, the lamb leg is roasted in an enclosed circular-shaped hollow pit, and charcoal is placed at the bottom of the pit as the heating source; the pit stays enclosed during the whole roasting process, which lasts for about 60 min, with roasting temperature around 250~280 °C. The material and structure of the roasting pit provide a good insulation and thermal radiation effect. The final Nang roasted lamb leg is shiny and golden brown in appearance; its outer layer is crisp while its inner part is juicy and tender. However, until now, there has been no comprehensive evaluation of the sensory qualities of roasted lamb processed with the traditional and special Nang roasting method.

Flavor is the most important sensory attribute affecting the qualities of roasted lamb [2], including aroma and taste, to which volatile and non-volatile compounds contribute, respectively [3]. Volatile compounds in meat products generally include aldehydes, alcohols, acids, ketones, esters, hydrocarbons and pyrazines compounds, which are generated by lipolysis, proteolysis, thiamine degradation and Millard reactions [4,5]. Nonvolatile compounds in meat products generally includes free amino acids, inorganic salts, flavor nucleotides and their degradation products, peptides, organic acids, etc. [6], which contribute to the umami taste, saltiness, sourness, sweetness and bitterness. In a previous study, the authors evaluated the effects of different roasting methods (microwave, charcoal, superheated steam and electric roasting) on the volatile and nonvolatile compounds of roasted lamb [7]. The results indicated that the lamb from electric roasting generated more nonvolatile compounds and had higher umami concentrations than lamb from microwave roasting and superheated steam roasting than those of lamb from microwave roasting and superheated steam roasting; meanwhile, the lamb from electric roasting and superheated steam roasting had higher odor activity values of volatile compounds than that of microwave roasting [7]. The volatile and nonvolatile compounds of roasted lamb varied significantly with various roasting methods. Therefore, as a unique and traditional roasting method, which is widely used in western China, the profiles of volatile and nonvolatile compounds of the Nang roasted lamb need to be comprehensively studied.

Gas chromatography mass spectrometry (GC-MS) is undoubtedly one of the key techniques that can be used for screening, identification and quantification of volatiles of complex food systems in a relatively short time, so it is widely used in flavor analysis of meat products [8]. Headspace solid phase microextraction (HS-SPME) integrates sampling, extraction, concentration and sample injection, which greatly improves the efficiency of detection and analysis of volatile compounds [9]. Many researchers have used HS-SPME combined with GC-MS to comprehensively evaluate the flavor compounds of roasted lamb [2,7,10], stir-fried pork slices [11], roasted beef [12] and smoked chicken [13]. Free amino acid and 5′-nucleotides are typical nonvolatile compounds in meat products. At present, free amino acid in meat products is commonly determined by an Amino Acid Automatic Analyzer [14]; 5′-nucleotides determination in meat products is always analyzed by high-performance liquid chromatography (HPLC) [15].

Therefore, the aims of this study were (1) to comprehensively evaluate the profiles of volatile and nonvolatile compounds in oyster cuts of roasted lamb with traditional Nang roasting at different processing stages; and (2) to reveal the volatile and nonvolatile markers in oyster cuts of traditional Nang roasted lamb at roasted stages. Eventually, we hope the results from this study might expand the research on the effects of different roasting methods on the flavor attributes of lamb meats, and provide theoretical support for the control of sensory attributes of roasted lamb with traditional Nang roasting.

## 2. Materials and Methods

### 2.1. Chemicals and Reagents

n-alkanes (C_7_-C_40_, 97%) standards were purchased from o2si Smart Solutions (Shanghai, China). 2-methyl-3-heptanone (99%), as GC-MS internal standard, was purchased from Dr. Ehrenstorfer GmbH (Beijing, China). 5′-nucleotide (99%) and free amino acids (99%) standards were purchased from Sigma-Aldrich Company (Shanghai, China).

### 2.2. Materials

A total of 36 male Xinjiang Kazak lamb were randomly selected, from the same genetics and the same feeding system (drylot feeding and same commercial diet), with an age of 7 months and carcass weight of about 24.9 ± 0.55 kg. These lambs were stunned, slaughtered and exsanguinated in accordance with the principles and guidelines established by the Animal Care and Use Committee of the institute of Food Science and Technology, Chinese Academy of Agricultural Sciences, at a local abattoir from Arman Food Group Co., Ltd., Xinjiang, China. After carcass cooling at 4 °C for 24 h, the left forelegs (about 1.5 kg each) were collected and immediately transported to a typical roasted meat restaurant in local Xinjiang by cold-chain logistics within one hour.

### 2.3. Sample Preparation

**Figure A1:**
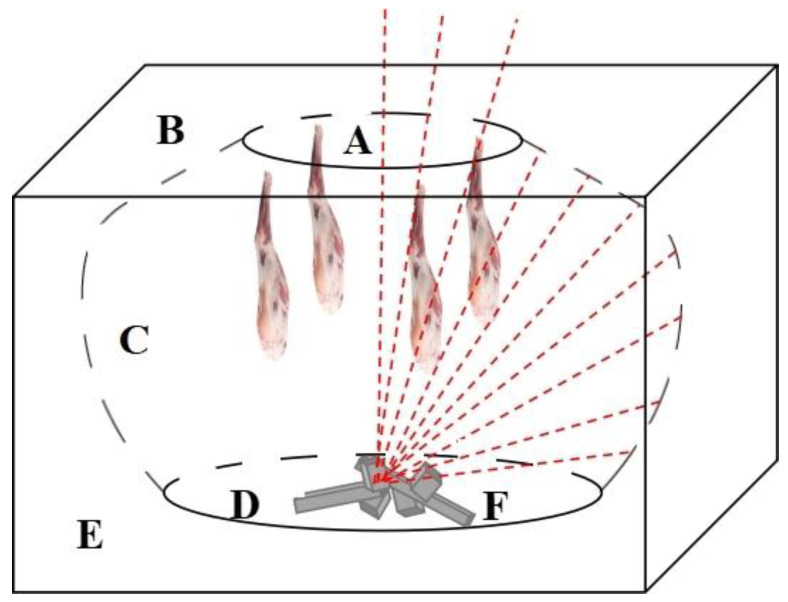
The diagrammatic drawing of Nang roasted lamb oyster cuts. A: Nang pit vent, B: platform, C: side of Nang pit, D: bottom of Nang pit, E: outer shell, F: heat source, red dotted line: thermal radiation.

A total of 36 left forelegs were equally divided into six groups. After removing the surface connective tissue and fat, six forelegs were randomly selected as the “fresh oyster cuts” group; the residual forelegs were divided into five groups and were all marinated with a 16% saline solution for 12 h at 4 °C. After that, one group (six forelegs) was served as the marinated oyster cuts group. The residual marinated forelegs samples were roasted by Xinjiang traditional Nang roasting in the roasted meat restaurant for 15, 30, 45 and 60 min, each contains six forelegs and designated as roasted-15, roasted-30, roasted-45 and roasted-60, respectively. The Nang roasting was carried out as described by the following procedure. Before roasting, charcoal was used to heat the inner wall of Nang pit. When the preheating temperature of the charcoal fire reached above 90 °C, the marinated samples were hung on the inner wall of the enclosed circular-shaped hollow pit, and began to be Nang roasted. During roasting processing, the roasting temperature was kept at 260 ± 10 °C, which was monitored using a 572-2 infrared thermometer (Fluke test instrument Co., Ltd., Shanghai, China). When the Nang roasting time reached 60 min, the core temperature of the lamb was 79 ± 2 °C. The detailed Nang roasting diagram can be seen in Figure A1 in Appendix A. After roasting for 15, 30, 45 and 60 min, the oyster cuts were immediately collected from the roasted forelegs and were wrapped in nylon/polyethylene (9.3 mL O_2_/m^2^/24 h, 0 °C, 0.19 mm thick, Magic Seal^®^, Guangdong, China). Then, the oyster cuts were put in liquid nitrogen and transported to a laboratory in Beijing by cold-chain logistics within five days. Analysis of volatile and nonvolatile compounds of these oyster cuts was performed after thawing at 3–5 °C for approximately 24 h when the core temperature of the oyster cuts reached −4 ± 1 °C.

### 2.4. Proximate Analysis

The moisture content, fat content and protein content of roasted oyster cuts were analyzed as described by the direct drying method, Soxhlet extraction method and the Kjeldahl method, respectively [16].

### 2.5. Volatile Compounds Analysis

#### 2.5.1. HS-SPME Analysis of the Volatile Compounds

The HS-SPME analyses carried out to extract the volatile compounds from the Nang roasted oyster cuts referred to the description of Liu et al. with slight modifications [17]. Briefly, 2 g minced roasted oyster cuts and 1.5 µL of internal standard (2-methyl-3-heptanone, 1.68 µg/μL) were added into a 20 mL headspace vial. The vial was equilibrated at 50 °C for 20 min, and an SPME fiber (65 μm PDMS/DVB extraction head, Supelco, Bellefonte, PA, USA) was exposed to the vial headspace for an additional 40 min at 50 °C. Finally, the extraction head was pulled out and desorbed in the GC inlet at 200 °C for 2 min.

#### 2.5.2. Qualitative and Quantitative Analysis of the Volatile Compounds

Volatile compounds of Nang roasted oyster cuts were analyzed according to the Liu et al. [17] method with minor modification using GC-MS (QP2010 Shimadzu, Kyoto, Japan) with a DB-WAX (30 mm × 0.25 mm × 0.25 μm). The carrier gas velocity was 1 mL/min. The oven temperature was maintained at 40 °C for 3 min, then heated to 120 °C at 5 °C/min, and finally heated to 200 °C at 10 °C/min, holding for 13 min. Ion source temperature was 200 °C. The scan mode was used to collect signals, and the scan range was 35–500 *m*/*z*. Volatile flavor compounds were qualitative identified by both the mass spectrometry database (NIST) search and linear retention index (LRI) compounds. The LRI was calculated by the retention time of *n*-alkanes (C_7_–C_40_) as in Equation (1):

(1) $$\mathrm{LRI}=100n+100(t_{x}-t_{n})/(t_{n+1}-t_{n})$$

where *t_x_* is the retention time of compound *x*; *t_n_* and *t_n +_*_1_ are the retention times of alkane *n* and alkane *n* + 1 (*t_n_* < *t_x_* < *t_n +_*_1_), respectively. *n* and *n +* 1 are the numbers of carbons in alkanes *n* and *n* + 1 closest to the retention time of compound *x*.

The contents of volatile flavor compounds were quantitatively calculated by comparing their peak areas with that of the internal standard.

#### 2.5.3. Odor Activity Value (OAV) of the Volatile Compounds

The OAVs of the volatile compounds were calculated as follows according to the method from Liu et al. [7], in which OAVs = C*i*/T*i*, where C*i* was the mass concentration of the volatile compounds *i* (µg/kg); T*i* was the olfactory threshold of the flavor compounds *i* (µg/kg), which were referenced from literatures on the website of https://www.vcf-online.nl/OFTVCompoundSearch.cfm, accessed on 27 November 2020. The OAV represents the contribution of each volatile compound to the general odor sense of Nang roasted oyster cuts. OAV > 1 indicates that the volatile compound has an important contribution to the odor sense of this product [18].

### 2.6. Determination of Nonvolatile Compounds

#### 2.6.1. Determination of Free Amino Acid

The determination of free amino acids contents in Nang roasted oyster cuts was based on the method of Liu et al. with slight modifications [18]. A measure of 20 mL of sulfosalicylic acid (3 g/100 mL) was mixed with 3 g of minced roasted oyster cuts, which were homogenized at 6000 r/min (40 s) and centrifuged with 10,000× *g*, for 15 min using the CR21N centrifuge (Hitachi Koki Co., Ltd., Ibaraki, Japan). The supernatant was added with 2 mL hexane, after which the supernatant was homogenized (3000 r/min, 60 s) using an IKA T18 digital ultra-turrax (IKA Group, Staufen, Germany) instrument. The supernatant was collected and dried with nitrogen using a TTL-DCII blower (Tongtailian Technology Development Co., Ltd., Beijing, China) at room temperature. After that, the dry matter was dissolved again with 1 mL 0.02 M HCl, and filtered with a 0.22 µm filter membrane. The free amino acids were determined using an L-8900 Amino Acid Automatic Analyzer (Hitachi Ltd., Tokyo, Japan). Free amino acids were identified and quantified by comparing the retention times and peak area of each amino acid standard (Sigma-Aldrich, St. Louis, MO, USA).

#### 2.6.2. Determination of Nucleotides

The contents of nucleotides in Nang roasted oyster cuts were analyzed by the method reported by Liu et al. [19] with slight modifications. A measure of 30 mL 5% perchloric acid was used to extract 10 g of ground roasted oyster cuts in an ice bath. The extracts were homogenized at 10,000× *g* (20 s × 2) with an Ultra-Turrax Disperser (Ika, German) in an ice bath condition. The homogenate solution was centrifuged (10,000× *g*, 10 min) by using the CR21N centrifuge (Hitachi Koki Co., Ltd., Ibaraki, Japan) at 4 °C. The pH of the supernatant was adjusted to 5.4 with 1 M NaOH, then filtered through a 0.45 μm filter. The filtrate was determined using high-performance liquid chromatography (Shimadzu Corporation, Japan) with the TSKgel ODS-80TM (5 µm, 4.6 mm × 250 mm) column. The column temperature was 30 °C. The ultraviolet detection wavelength was 254 nm. The flow rate was 0.8 mL/min and the injection volume was 10 μL. The eluent A was 0.05 M potassium dihydrogen phosphate buffer at pH 5.4 and eluent B was methanol (HPLC grade). The binary mobile phase was used for gradient elution separation, and the detection time was 25 min. The gradient elution program of A/B was conducted as follows: 99%/1% (0–15 min), 90%/10% (16–20 min), 99%/1% (21–25 min). Nucleotides were identified and quantified by comparing the retention times and peak areas of each nucleotide standard (Sigma-Aldrich, St. Louis, MO, USA).

#### 2.6.3. Taste Activity Value (TAV) of the Nonvolatile Compounds

The TAV of the nonvolatile compounds is calculated as the ratio of the concentration of nonvolatile compounds in Nang roasted oyster cuts to their taste threshold values [20]. The taste threshold value was obtained from the literature of Zou et al. [14]. The TAV represents the contribution of each nonvolatile compound to the general taste sense of Nang roasted oyster cuts. TAV > 1 indicate the nonvolatile compound has a great contribution to the taste sense of this product [7].

#### 2.6.4. Equivalent Umami Concentration (EUC) of Nonvolatile Compounds

The EUC is regarded as the concentration of monosodium glutamate (MSG, g/100 g) equivalent to the umami intensity generated by 5′-nucleotides (5′-GMP, 5′-IMP, 5′-AMP) and umami amino acids (glutamic acid and aspartic acid). The EUC of nonvolatile compounds was reported by Yamaguchi et al. [21], and was calculated by the following formula:

(2) $$\mathrm{EUC}=\Sigma aibi+1218\left(\Sigma aibi\right)\Sigma ajbj_{\;}$$

The unit of EUC was g MSG/100 g and 1218 is a synergistic constant. a*_i_* and b*_i_* were the concentration of umami amino acids (aspartic acid or glutamic acid) (g/100 g) and the relative umami coefficients (RUC) for the umami amino acid compared to MSG (glutamic acid: 1, aspartic acid: 0.077), respectively. a*_j_* and b*_j_* were the concentrations of taste 5-nucleotides (5′-IMP, 5′-AMP, 5′-GMP) (g/100 g), and the RUC of taste 5′-nucleotides (5′-AMP: 0.18; 5′-IMP: 1; 5′-GMP: 2.3), respectively.

### 2.7. Statistical Analysis

All data were analyzed by one-way ANOVA using a general linear model and Tukey’s test using SPSS v. 19.0 software (IBM Corporation, Chicago, IL, USA). The OAV of volatile compounds and the TAV of nonvolatile compounds are presented with a heatmap, and were performed with Origin (version 2016, Origin Lab, Hampton, MA, USA). To distinguish (non)volatile compounds of Nang roasted oyster cuts at different processing stages, the orthogonal partial least square discriminant analysis (OPLS-DA) model was used. The OPLS-DA model was performed with SIMCA software version14.1 (Umetrics, Umea, Sweden). Among them, the (non)volatile compounds were selected as X-variable and Nang roasting stages were selected as Y-variable. The results are shown as the mean ± standard deviations of six replicates.

## 3. Results and Discussion

### 3.1. Changes of Proximate Compositions in Nang Roasted Oyster Cuts at Different Processing Stages

The contents of proximate compositions in Nang roasted oyster cuts are presented in Table 1. The moisture content of the samples decreased significantly from that in a fresh sample to that in roasted-60 (*p* < 0.05). Long time heating gives rise to more moisture loss. The protein content increased within 30 min of roasting from that in a fresh sample to that in roasted-30, then decreased significantly to that in roasted-60 (*p* < 0.05). Extended thermal treatment could lead to protein degradation and modification in meat products [22], which most likely results in decreased protein content at the end of the roasting process. As a result of protein degradation, more flavor precursors such as free amino acids would be generated [23], which could contribute to the aroma formation of roasted lamb. The fat content significantly decreased from that in a fresh sample to that in roasted-60, which might be the consequence of degradation and oxidation of lipid along with roasting time [18], and lipid oxidation played an important effect on the formation of flavor compounds in the meat [24].

### 3.2. Changes of Volatile Compounds in Nang Roasted Oyster Cuts at Different Processing Stages

#### 3.2.1. The Profiles of Volatile Compounds

**Figure 1:**
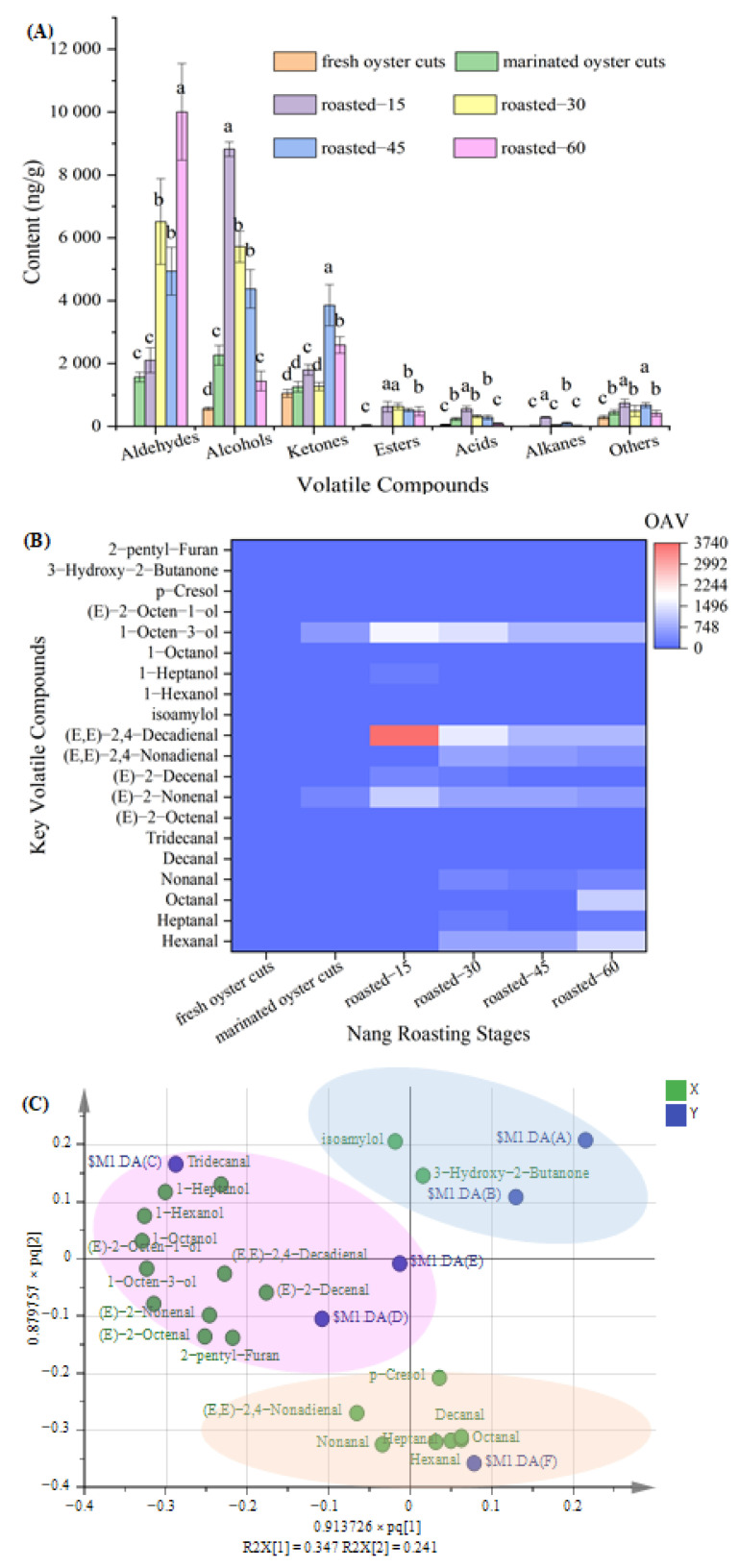
Distribution of volatile compounds groups (**A**), heatmap analysis of volatile compounds with odor activity value (OAV) > 1 (**B**) and orthogonal partial least square discriminant analysis (OPLS-DA) based on volatile compounds with OAV > 1 in Nang roasted oyster cuts (**C**). $M1.DA(A), fresh oyster cuts; $M1.DA(B), marinated oyster cuts; $M1.DA(C), $M1.DA(D), $M1.DA(E) and $M1.DA(F) means roasted-15, roasted-30, roasted-45 and roasted-60, respectively. X: volatile flavor compounds (OAV > 1), Y: Nang roasting stages. Different lowercase letters in the same row indicate that there is significant difference (*p* < 0.05).

The types and contents of volatile compounds in the product are presented in Table 2. A total of 51 volatile compounds were detected, including aldehydes (16), alcohols (15), ketones (4), esters (1), acids (3), hydrocarbons (5) and some other compounds (7), which was consistent with previous studies [2,7]. Results from Figure 1A indicated that the total content of volatile compounds showed an upward trend from 2004.51 ng/g in fresh oyster cuts to 15,055.85 ng/g in roasted-60. The total content of aldehydes was the highest, followed by alcohols and then ketones. Ketones mainly come from the Maillard reaction, the thermal oxidation of lipid and amino acid degradation [13]. One ketone was detected in fresh oyster cuts with the value of 1050.67 ng/g, while 3 types of ketones in roasted-45 was identified with the value of 3855.86 ng/g, but then decreased to 2590.14 ng/g in roasted-60. The types and contents of alcohols increased significantly from 8 types and 570.59 ng/g in fresh oyster cuts to 13 types and 8818.7 ng/g in roasted-15 (*p* < 0.05), respectively; however, it gradually decreased to 4 types and 1445.06 ng/g in roasted-60. Alcohols come from the oxidation and degradation of lipids, but they have less effect on the aroma of meat due to their higher odor thresholds [12]. Aldehydes were not detected in fresh oyster cuts; with extended roasting time, the types and contents of the aldehydes increased and reached their maximum values of 12 types and 15,125.98 ng/gin roasting-60. The odor threshold of aldehydes is significantly lower than that of alcohols and ketones (*p* < 0.05), and they have a strong influence on the aroma of meat products [25]. Regardless of their contents, types and values of threshold, aldehydes are the dominant profiles of volatile compounds in Nang roasted oyster cuts.

#### 3.2.2. The Key Volatile Compounds

The OAV was used to evaluate the contribution of volatile compounds to Nang roasted oyster cuts. Figure 1B indicated the volatile compounds with OAV > 1 (key volatile compounds) in different samples. In fresh oyster cuts, the main volatile compounds were 1-octen-3-ol, with a 96.50% contribution rate to the total OAV. In marinated oyster cuts, the main volatile compounds were hexanal, (E)-2-nonenal, nonanal and 1-octen-3-ol, with a 86.92% cumulative contribution rate to the total OAV. In roasted-15, (E, E)-2,4-decadienal, (E)-2-nonenal and 1-octen-3-ol were the main volatile compounds, and their cumulative contribution rate was 88.66%. In roasted-30, the main volatile compounds were hexanal, (E)-2-nonenal, (E, E)-2,4-decadienal, (E, E)-2,4-nonadienal and 1-octen-3-ol, with a 85.14% cumulative contribution rate. In roasted-45, the main volatile compounds were hexanal, (E, E)-2,4-decadienal, (E)-2-nonenal, 1-octen-3-ol and (E, E)-2,4-nonadienal, with a 91.61% cumulative contribution rate. In roasted-60, the main volatile compounds were hexanal, (E, E)-2,4-decadienal, octanal, (E, E)-2,4-nonadienal, (E)-2-nonenal and 1-octen-3-ol, and their cumulative contribution rate was 89.25%.

Aldehydes are generally produced by lipid oxidation and degradation [14]. The OAV of hexanal, octanal and nonanal showed an upward trend during the whole roasting process. The content of hexanal with 0~5728.83 ng/g was the highest, and it was also reported with high content in charcoal roasted lamb [7]. Hexanal generally comes from the oxidation of n-6 polyunsaturated fatty acid [26], and nonanal and octanal come from the oxidation of n-9 polyunsaturated fatty acid [27]. These aldehydes have a pleasant fruity flavor even at low concentration [28,29] and might have contributed to the roasted lamb aroma. The OAV of (E)-2-nonenal, (E, E)-2,4-decadienal and (E, E)-2,4-nonadienal significantly increased from fresh to roasted-30 (*p* < 0.05), and after that, decreased until roasted-60. The OAV of (E, E)-2,4-decadienal decreased, which was consistent with the result of Liu et al. [7]. Extended roasting could cause slight aroma loss [30]. (E, E)-2,4-decadienal and (E)-2-nonenal mainly contribute to the fatty aroma [2,14], and also play a critical role in the overall aroma of the roasted samples. Alcohols also come from the oxidation and degradation of lipids [12]. The OAV of 1-octen-3-ol significantly increased from fresh to roasted-15, and after that, decreased until roasted-60 (*p* < 0.05). As a product of lipid oxidation and degradation, 1-octen-3-ol might be oxidized to aldehydes and esters [1], which most likely resulted in the decrease of 1-octen-3-ol in the late stage of roasting. In this study, because of its high OAV, 1-octen-3-ol, with a mushroom aroma [31], might be an important aroma contributor to the roasted samples. The results of OAV changes of volatile compounds during roasting suggested that hexanal, nonanal, octanal, (E, E)-2,4-decadienal, (E)-2-nonenal, 1-octen-3-ol and (E, E)-2,4-nonadienal were the key volatile compounds and aroma contributors to Nang roasted oyster cuts.

#### 3.2.3. Changes of Key Volatiles Compounds in Nang Roasted Oyster Cuts at Different Processing Stages

In order to understand the differences of volatile compounds at different processing stages, 20 kinds of volatile compounds (OAV > 1) were analyzed by OPLS-DA (Figure 1C). A clear separation of the projection was observed at different processing stages (R^2^X = 0.916, R^2^Y = 0.811, and Q^2^ = 0.62). The key volatile compounds were significantly influenced by roasting time. The projections of the fresh and marinated samples were located in the positive direction of the t_1_ and t_2_, far away from that of the roasted samples. With a roasting time from 15 to 60 min, the projection of roasted samples gradually moved from the positive direction to the negative direction of the t_2_. The results indicated that the key volatile compounds of different processing stages were significantly different. Isoamylol and 3-hydroxy-2-butanone were positively related to OPLS-component t_2_ and could distinguish fresh and marinated oyster cuts from roasted samples. 1-octen-3-ol, (E)-2-nonenal and (E, E)-2,4-decadienal were located in the negative direction of t_1_, and hexanal, (E, E)-2,4-nonadienal, octanal and nonanal were located in the negative direction of t_2_, which primarily distinguished roasted-60 from roasted-15, roasted-30 and roasted-45 samples. These results were also consistent with the results of HS-SPME/GC-MS.

### 3.3. Changes of Nonvolatile Compounds in Nang Roasted Oyster Cuts at Different Processing Stages

#### 3.3.1. The Profiles of Free Amino Acids

A total of 17 free amino acids were detected in roasted oyster cuts (Table 3), including 2 umami amino acids, 5 sweet amino acids, 9 bitter amino acids and one other free amino acid. The total contents of umami amino acids were the highest, followed by sweet amino acids, then the bitter amino acids. The contents of total free amino acids and umami amino acids in roasted samples significantly increased from those in fresh samples to those in marinated samples (*p* < 0.05), and after that, decreased to those in roasted-60 samples, respectively. The content of free amino acids increased significantly after marinating (*p* < 0.05), which may be due to the degradation of protein under the action of proteolytic enzyme and aminopeptidase during marinating [32]. Free amino acids decreased during the late period of roasting might be closely related with the formation of flavor compounds, because under high thermal treatment, the free amino acids in meat could convert into small-molecule volatile compounds including aldehydes, ketones, esters, furans and alcohols through the Maillard reaction [14,24], which was also consistent with the changes of the aldehydes, alcohols and ketones in Nang roasted samples. The contents of sweet amino acids and bitter amino acids in roasted samples decreased continuously during the whole processing stage from those in fresh oyster cuts to those in roasted-60 samples, respectively. In terms of the changes of the content of free amino acids, umami amino acids and sweet amino acids are the dominant nonvolatile compounds in Nang roasted oyster cuts.

#### 3.3.2. The Key Free Amino Acids

**Figure 2:**
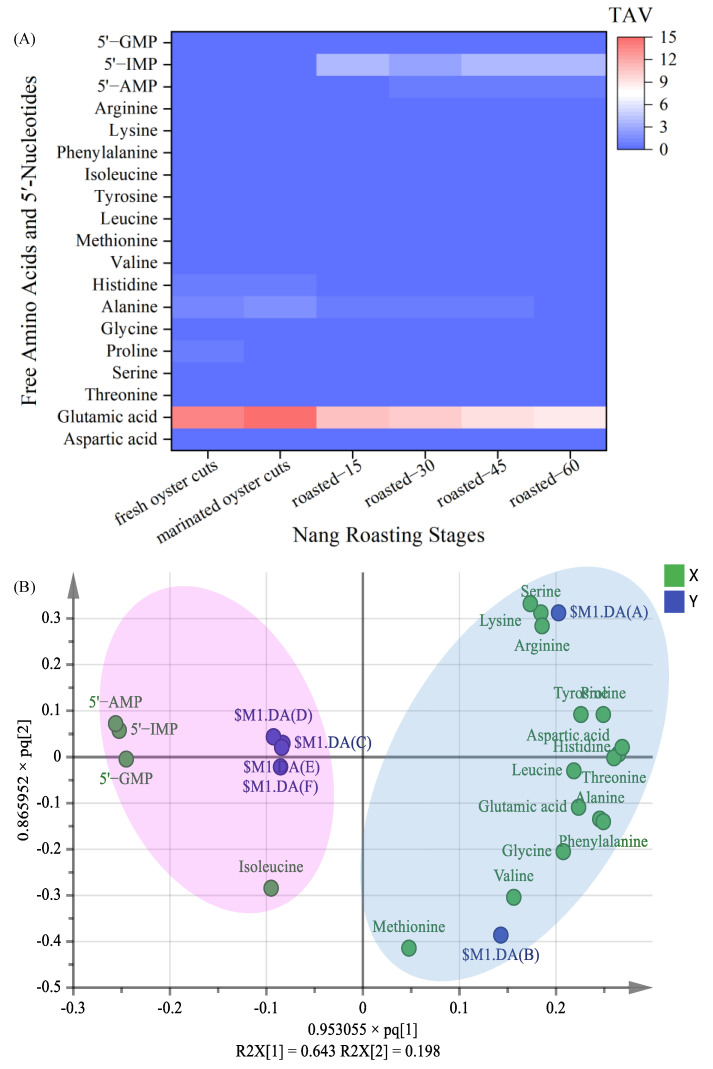
Taste activity value (TAV) of nonvolatile compounds (**A**) and orthogonal partial least square discriminant analysis (OPLS-DA) based on nonvolatile compounds in Nang roasted oyster cuts (**B**). $M1.DA(A), fresh oyster cuts; $M1.DA(B), marinated oyster cuts; $M1.DA(C), $M1.DA(D), $M1.DA(E) and $M1.DA(F) means roasted-15, roasted-30, roasted-45 and roasted-60, respectively. X: nonvolatile compounds, Y: Nang roasting stages.

The contribution of free amino acids to the taste of roasted oyster cuts was not only related to their content but also to their TAV (Figure 2A). The TAV of glutamic acid during the whole processing was much higher than the other free amino acids, which was 13.88 in fresh oyster cuts, and increased significantly to 15.38 in marinated samples, then after that, gradually decreased to 8.83 in roasted-60 samples. The TAV of alanine ranks second; it was 1.40 and 1.68 in fresh and marinated samples, respectively. After roasting, the TAV of alanine was less than 1. Kawai et al. [33] found that alanine could be combined with glutamic acid to enhance umami taste. Qi et al. [20] and Zou et al. [14] also reported that glutamic acid and alanine were the flavor amino acids of chicken soup and spiced beef. Previous report [34] indicated that when alanine and glycine were in contact with sweet substance receptors, they could produce a strong sweet taste and counteract salty and bitter tastes. Chen et al. [35] reported that alanine and glycine were the main sweet taste contributors in crab meat. In terms of the TAV changes of free amino acids, glutamic acid and alanine were the key free amino acids or taste contributor to Nang roasted oyster cuts.

#### 3.3.3. The Profiles of 5′-Nucleotides

The changes of 5′-nucleotides, including GMP, IMP, AMP, ADP, Hx and I in Nang roasted oyster cuts at different processing stages are shown in Table 4. Among them, the content of 5′-inosine monophosphate (5′-IMP) was the highest, accounting for 44.12-78.80% of total flavor nucleotides, followed by inosine (I), then hypoxanthine (Hx). The contents of flavor 5′-nucleotides and 5′-IMP in roasted samples gradually increased during the whole processing from those in fresh oyster cuts to their maximum contents, those in roasted-15 samples, then remained relatively stable (*p* > 0.05) until roasted-60. The content of I gradually increased and reached their maximum values in roasted-60 samples (*p* < 0.05). The contents of Hx increased significantly from that in fresh samples to that in marinated samples, and after that, decreased significantly to that in roasted-60 samples (*p* < 0.05). Under the catalysis of phosphokinase, 5′-ADP was decomposed into 5′-AMP, then 5′-AMP was dehydrogenated to 5′-IMP [36]. Part of 5′-IMP forms I under the effect of phosphokinase. These reactions might be the reason for the increase in content of 5′-IMP and I [36]. The 5′-nucleotides in meat and meat products are an important contributor to meat flavor and have been widely used as an ingredient to enhance the flavor in meat products [14]. Mateo et al. [37] pointed out that the inosine produced a bitter taste, and hypoxanthine did not contribute to any taste response during the ripening of chorizo.

#### 3.3.4. The Key 5′-Nucleotides

In terms of the TAV of 5′-nucleotides (Figure 2A), the values of 5′-IMP were the highest; it was the key 5′-nucleotide in roasted oyster cuts, and its value increased significantly from 0.27 in fresh oyster cuts to 3.93 in roasted-15 samples (*p* < 0.05), and after that, remained stable until roasted-60 within the range of 3.05~3.93. The result suggested that Nang roasting treatment increased the concentration of umami taste of roasted samples compared with fresh and marinated samples. Liu et al. [19] reported that 5′-IMP was the main taste contributor in Nanjing cooked duck. Zou et al. found that 5′-IMP was the primary flavor nucleotide in spiced beef [14]. Kawai et al. [33] pointed out that the interaction of IMP with sweet amino acids, such as alanine, glycine and serine, increased the intensity of the umami taste. Although the TAV of 5′-AMP and 5′-GMP in roasted oyster cuts were both lower than 1, a synergistic effect exists between 5′-IMP and 5′-AMP to enhance the umami taste [3]. In addition, GMP is a stronger flavor enhancer contributing to a meat flavor [3].

The synergistic effects of three taste nucleotides (5′-GMP, 5′-IMP and 5′-AMP) with free amino acids and inorganic salt ions give meat products a unique umami taste [21]. The synergistic effects of 5′-nucleotides with glutamic acid and aspartic acid were evaluated by EUC (Table 4). For the EUC value, there was no significant difference between fresh and marinated samples. The EUC increased significantly from that of in marinated oyster cuts to that of in roasted-15 samples, then remained relatively stable (*p* > 0.05) until roasted for 60 min. The EUC increased significantly in roasted-15 samples, which was due to the high content of 5′-IMP and glutamic acid in these samples. The result indicated that there was a synergistic interaction of umami components during Nang roasting: that was to say, Nang roasting increased the umami intensity of oyster cuts. Roasting significantly increased the umami taste intensity of samples. In terms of the changes of TAV and EUC, 5′-IMP was also the key nonvolatile compounds or taste contributor to Nang roasted oyster cuts.

#### 3.3.5. Changes of the Key Nonvolatile Compounds

In order to further understand the differences of nonvolatile compounds at different processing stages, 19 nonvolatile compounds (16 free amino acids and 3 5′-nucleotides) were performed by OPLS-DA. The key taste compounds were analyzed by OPLS-DA (Figure 2B). A clear separation of the projection was observed at different processing stages (R^2^X = 0.908, R^2^Y = 0.617, and Q^2^ = 0.429). The fresh and marinated samples were far from the others, indicating that the taste of fresh and marinated samples was significantly different from the others. The glutamic acid and alanine were located in the positive direction of t_1_ and the negative direction of t_2_, and 5′-IMP were located in the negative direction of t_1_, which primarily distinguished roasted samples from fresh and marinated ones.

## 4. Conclusions

As a traditional roasting method with a history of thousands of years, Nang roasting exerts a unique flavor to roasted lamb samples. Comprehensive evaluation of volatile and nonvolatile compounds of Nang roasted samples were performed. The volatile and nonvolatile compounds varied significantly at different roasting stages. Aldehydes were the dominant volatile compounds, followed by alcohols, then ketones. Umami amino acids were the main nonvolatile compounds, followed by sweet amino acids, then bitter amino acids. The OAV and TAV were calculated to evaluate the contribution of volatile compounds and nonvolatile compounds to sensory attributes of Nang roasted oyster cuts, respectively. Hexanal, (E)-2-nonenal, octanal, (E, E)-2,4-nonadienal, nonanal, 1-octen-3-ol and (E, E)-2,4-decadienal were the key volatile compounds, and glutamic acid, alanine and 5′-IMP were the key nonvolatile compounds. A total of 20 kinds of volatile compounds (OAV > 1) and 19 kinds of nonvolatile compounds were analyzed by OPLS-DA. A clear separation of the projection was observed at different processing stages. For fresh and marinated oyster cuts, isoamylol and 3-hydroxy-2-butanone were important flavor contributors, and glutamic acid and alanine were important taste contributors. For roasted-15, 30 and 45 ones, (E)-2-nonenal, 1-octen-3-ol and (E, E)-2,4-decadienal were important flavor contributors. For roasted-60 ones, hexanal, nonanal, (E, E)-2,4-nonadienal and octanal were important flavor contributors, while 5′-IMP was the most important taste contributor in roasted oyster cuts.

## Figures and Tables

**Table 1:** Changes of physicochemical properties in lamb oyster cuts during Nang roasting (dry matter basis).

Physicochemical Properties	Nang Roasting Stages					
	Fresh Oyster Cuts	Marinated Oyster Cuts	Roasted-15	Roasted-30	Roasted-45	Roasted-60
Moisture/%	72.90 ± 2.89 ^a^	73.03 ± 1.31 ^a^	67.46 ± 1.52 ^b^	64.27 ± 1.14 ^c^	60.36 ± 1.71 ^d^	59.05 ± 1.28 ^d^
Protein/%	69.95 ± 1.33 ^b^	65.31 ± 0.61 ^d^	71.94 ± 2.22 ^a^	72.06 ± 1.52 ^a^	67.35 ± 1.09 ^cd^	69.65 ± 2.31 ^b^
Fat/%	18.12 ± 0.64 ^a^	18.08 ± 0.55 ^a^	15.44 ± 1.52 ^b^	15.09 ± 0.94 ^b^	14.94 ± 1.46 ^c^	10.41 ± 0.48 ^d^

Different lowercase letters in the same row indicate that there is significant difference (*p* < 0.05).

**Table 2:** Contents of the volatile compounds in lamb oyster cuts during Nang roasting (ng/g).

Compounds	Lri ^a^		Identification ^d^	Ions (*m*/*z*)	Nang Roasting Stages					
	Literature ^b^	Calculated ^c^			Fresh Oyster Cuts	Marinated Oyster Cuts	Roasted-15	Roasted-30	Roasted-45	Roasted-60
Aldehydes	-	-	-	-	-	-	-	-	-	-
Hexanal	1074	1077	MS + LRI	44	ND	512.81 ± 62.59 c	367.48 ± 161.23 c	3025.93 ± 163.3 b	3250.66 ± 269.46 b	5728.83 ± 1202.9 a
Heptanal	1174	1178	MS + LRI	70	ND	161.81 ± 8.43 b	ND	522.17 ± 87.42 a	ND	435.07 ± 107.89 a
Octanal	1277	1280	MS + LRI	41	ND	ND	ND	ND	ND	853.20 ± 249.03
Nonanal	1385	1384	MS + LRI	57	ND	711.44 ± 112.43 c	676.48 ± 65.64 c	2275.44 ± 371.36 a	1104.52 ± 234.80 b	2392.88 ± 727.74 a
Decanal	1483	1493	MS + LRI	41	ND	ND	ND	ND	ND	75.9 ± 13.51 a
Tridecanal	1824	1812	MS + LRI	57	ND	ND	104.65 ± 38.26 a	ND	ND	ND
2-Undecenal	1755	1746	MS + LRI	70	ND	ND	ND	ND	120.13 ± 0.43 a	ND
Tetradecanal	1931	1920	MS + LRI	57	ND	ND	ND	ND	ND	29.78 ± 7.34 a
Hexadecanal	2141	2134	MS + LRI	57	ND	ND	ND	ND	ND	35.57 ± 6.41 a
Heptadecanal	2249	2247	MS + LRI	82	ND	ND	ND	21.95 ± 12.24 a	ND	ND
(E)-2-Octenal	1416	1419	MS + LRI	41	ND	98.57 ± 0.86 c	272.52 ± 47.24 a	247.39 ± 83.16 a	174.91 ± 15.74 a b	151.60 ± 61.84 b
(E)-2-Nonenal	1535	1525	MS + LRI	41	ND	67.37 ± 1.41 c	282.33 ± 71.79 a	172.58 ± 75.42 b	177.36 ± 51.07 b	133.14 ± 14.17 b
(E)-2-Decenal	1634	1636	MS + LRI	43	ND	ND	142.60 ± 9.20 a	79.75 ± 14.65 b	ND	49.57 ± 17.23 b
Benzaldehyde	1508	1507	MS + LRI	77	ND	16.45 ± 2.97 a	ND	ND	ND	ND
(E, E)-2,4-Nonadienal	1686	1692	MS + LRI	81	ND	ND	ND	65.89 ± 15.84 a	50.19 ± 4.74 b	49.78 ± 7.08 b
(E, E)-2,4-Decadienal	1804	1801	MS + LRI	81	ND	ND	261.21 ± 35.65 a	105.12 ± 19.17 b	62.73 ± 14.06 c	69.81 ± 16.66 c
Alcohols	-	-	-	-	-	-	-	-	-	-
isoamylol	1210	1217	MS + LRI	55	254.57 ± 36.22 c	756.99 ± 175.33 a	436.68 ± 69.39 a b	ND	ND	ND
1-Pentanol	1252	1251	MS + LRI	42	15.20 ± 8.22 c	58.10 ± 15.49 c	479.89 ± 176.27 a	348.63 ± 66.75 b a	222.77 ± 17.65 b	169.02 ± 30.61 b c
1-Hexanol	1358	1353	MS + LRI	56	64.70 ± 5.44 d	204.91 ± 57.69 d	3130.19 ± 1363.53 a	2524.30 ± 569.71 b c	1616.83 ± 293.23 c	ND
1-Heptanol	1456	1455	MS + LRI	70	ND	152.66 ± 19.93 c	476.22 ± 137.41 a	263.20 ± 95.07 b	325.37 ± 67.09 a b	ND
1-Octanol	1554	1557	MS + LRI	56	21.86 ± 0.53 d	80.97 ± 14.61 d	781.59 ± 284.57 a	372.65 ± 153.69 b	367.63 ± 155.73 b	194.47 ± 63.05 c
1-Nonanol	1661	1662	MS + LRI	55	ND	ND	496.34 ± 186.77 a	ND	302.89 ± 101.73 b	ND
1-Octen-3-ol	1451	1450	MS + LRI	57	112.03 ± 6.43e	535.24 ± 157.33 d	1689.58 ± 632.75 a	1394.20 ± 199.32 a b	876.69 ± 62.15 b c	915.46 ± 235.34 b c
Benzyl alcohol	1866	1868	MS + LRI	79	2.00 ± 0.87 b	ND	12.76 ± 5.76 a	ND	ND	ND
Phenylethyl alcohol	1909	1906	MS + LRI	91	17.54 ± 8.39 a	24.75 ± 8.17 a	ND	ND	ND	ND
Eucalyptol	1204	1212	MS + LRI	43	ND	ND	172.43 ± 42.48 a	49.39 ± 11.86 b	36.08 ± 11.23 b	ND
2,3-Butanediol	1570	1573	MS + LRI	45	82.69 ± 0.28 d	208.04 ± 9.46 c	561.34 ± 74.25 a	494.15 ± 88.12 b	453.92 ± 234.05 b	ND
(E)-2-Octen-1-ol	1617	1611	MS + LRI	57	ND	106.30 ± 8.83 d	463.70 ± 129.76 a	272.49 ± 42.86 b	171.70 ± 8.02 c	166.11 ± 54.00 c
(E)-2-Nonen-1-ol	1722	1713	MS + LRI	57	ND	ND	32.47 ± 18.48 a	ND	ND	ND
Terpinen-4-ol	1595	1598	MS + LRI	71	ND	78.33 ± 23.51 a	85.51 ± 16.62 a	ND	ND	ND
alpha. -Terpineol	1688	1695	MS + LRI	59	ND	58.97 ± 21.35 a	ND	ND	ND	ND
Ketones	-	-	-	-	-	-	-	-	-	-
3-Hydroxy-2-Butanone	1275	1278	MS + LRI	45	1050.67 ± 205.78 c	1121.36 ± 294.36 c	1283.36 ± 103.87 b	1235.51 ± 98.14 b	2597.47 ± 207.43 a	ND
3-Octen-2-one	1414	1410	MS + LRI	55	ND	ND	ND	36.57 ± 11.66 b	ND	70.74 ± 25.62 a
2,3-Octanedione	1325	1325	MS + LRI	43	ND	138.63 ± 8.40 d	501.72 ± 97.99 c	ND	1244.92 ± 629.25 b	2508.95 ± 804.81 a
2-Undecanone	1325	1325	MS + LRI	58	ND	ND	15.95 ± 5.46 a	ND	13.47 ± 2.24 a	10.45 ± 2.66 a
Esters	-	-	-	-	-	-	-	-	-	-
n-Caproic acid vinyl ester	-	-	MS	43	37.10 ± 1.37 c	ND	628.57 ± 67.99 a	639.57 ± 108.82 a	522.29 ± 142.11 b	483.70 ± 19.01 b
Acids	-	-	-	-	-	-	-	-	-	-
Butanoic acid	1663	1666	MS + LRI	60	54.31 ± 13.01 c	108.43 ± 31.83 a b	133.81 ± 2.36 a	81.90 ± 16.41 b	67.84 ± 12.36 c	ND
Hexanoic acid	1849	1839	MS + LRI	60	ND	125.60 ± 23.46 c	423.90 ± 84.10 a	242.26 ± 46.37 b	218.23 ± 30.96 b	85.64 ± 20.42 c
Octanoic acid	2060	2056	MS + LRI	60	ND	7.85 ± 2.56 a	ND	ND	ND	ND
Alkanes	-	-	-	-	-	-	-	-	-	-
1-Pentadecene	1545	1545	MS + LRI	41	ND	ND	ND	ND	21.84 ± 5.38 a	ND
Styrene	1252	1250	MS + LRI	104	ND	29.05 ± 9.57 b	ND	41.32 ± 7.66 ab	52.30 ± 10.54 a	ND
Dodecane	-	-	MS	57	ND	ND	84.23 ± 12.69 a	ND	ND	ND
Tetradecane	-	-	MS	57	ND	ND	123.99 ± 24.92 a	ND	ND	ND
Heptadecane	-	-	MS	57	ND	ND	87.10 ± 12.44 a	ND	31.99 ± 7.09 b	27.45 ± 11.11 b
Others	-	-	-	-	-	-	-	-	-	-
Phenol	1992	1994	MS + LRI	94	ND	ND	ND	ND	163.16 ± 14.96 a	136.06 ± 13.51 b
p-Cresol	2068	2059	MS + LRI	107	ND	ND	ND	ND	62.02 ± 5.72 a	65.02 ± 18.17 a
Anethole	1818	1817	MS + LRI	148	ND	ND	39.14 ± 9.40 a	15.86 ± 8.08 ab	11.39 ± 1.60 b	ND
Biphenyl	1981	1981	MS + LRI	154	ND	ND	ND	17.69 ± 7.22 b	37.47 ± 3.40 a	ND
2-pentyl-Furan	1230	1216	MS + LRI	81	ND	55.03 ± 5.07 b	138.53 ± 27.65 a	91.33 ± 18.89 ab	93.14 ± 36.30 ab	109.95 ± 32.26 ab
methoxy-phenyl-Oxime	-	-	MS	133	291.84 ± 53.06 c	385.81 ± 115.95 b	529.29 ± 164.69 a	371.59 ± 52.09 b	310.00 ± 69.05 b	107.70 ± 24.25 cd
1,2,3,4-tetramethyl-Benzene	1430	1424	MS + LRI	119	ND	11.59 ± 5.09 a	32.55 ± 17.56 a	ND	ND	ND

Different lowercase letters in the same row indicate that there is significant difference (*p* < 0.05). ^a^ Linear retention index. ^b^ Reported data. ^c^ Calculated data based on *n*-alkanes (C_7_–C_40_). ^d^ Means of identification: MS, mass spectrum comparison using NIST libraries; LRI, linear retention index compared with literature values. ND: volatile compounds not detected. “-”: not reported in the literature.

**Table 3:** Changes of free amino acid content in lamb oyster cuts during Nang roasting (dry matter basis).

Free Amino Acid(mg/100 g)	Nang Roasting Stages						Taste Threshold (mg/100 g)
	Fresh Oyster Cuts	Marinated Oyster Cuts	Roasted-15	Roasted-30	Roasted-45	Roasted-60	
Aspartic acid	23.28 ± 2.61 ^a^	19.23 ± 1.02 ^a^	2.87 ± 0.57 ^b^	2.79 ± 0.52 ^b^	2.33 ± 0.59 ^b^	1.98 ± 0.29 ^b^	100
Glutamic acid	416.35 ± 11.99 ^b^	461.39 ± 11.22 ^a^	329.74 ± 13.71 ^b^	318.87 ± 12.57 ^bc^	282.82 ± 10.19 ^c^	264.89 ± 1.66 ^c^	30
∑UAA	439.63 ± 9.61 ^b^	480.62 ± 9.12 ^a^	332.61 ± 12.57 ^c^	321.66 ± 10.27 ^c^	285.15 ± 5.19 ^d^	266.87 ± 4.62 ^d^	/
Threonine	22.42 ± 2.96 ^a^	19.99 ± 1.15 ^a^	7.39 ± 0.68 ^b^	6.92 ± 1.51 ^b^	6.78 ± 0.51 ^b^	5.46 ± 1.00 ^b^	260
Serine	19.98 ± 2.43 ^a^	5.17 ± 0.26 ^b^	5.72 ± 0.37 ^b^	6.41 ± 0.97 ^b^	5.96 ± 0.40 ^b^	5.34 ± 0.81 ^b^	150
Proline	190.23 ± 7.26 ^a^	115.81 ± 9.67 ^b^	41.30 ± 1.88 ^c^	27.99 ± 1.65 ^c d^	21.88 ± 11.05 ^d^	14.33 ± 1.92^e^	300
Glycine	7.92 ± 0.57 ^b^	15.39 ± 1.66 ^a^	1.95 ± 0.39 ^c^	1.81 ± 0.72 ^c^	2.23 ± 0.93 ^c^	3.19 ± 0.39 ^c^	130
Alanine	84.16 ± 6.02 ^b^	100.94 ± 5.53 ^a^	52.74 ± 1.79 ^c^	40.46 ± 2.19 ^d^	39.79 ± 1.14 ^d^	30.60 ± 1.75 ^d^	60
∑SAA	324.71 ± 10.56 ^a^	257.3 ± 6.57 ^b^	109.1 ± 3.83 ^c^	83.59 ± 4.57 ^d^	76.64 ± 2.43 ^d^	58.92 ± 5.35^e^	/
Valine	4.74 ± 0.26 ^b^	16.47 ± 1.30 ^a^	2.02 ± 0.48 ^b^	1.82 ± 0.17 ^b^	2.00 ± 0.44 ^b^	1.99 ± 0.38 ^b^	40
Methionine	0.21 ± 0.05 ^d^	3.95 ± 0.19 ^a^	1.16 ± 0.46 ^c^	0.65 ± 0.20 ^d^	1.40 ± 0.03 ^bc^	1.89 ± 0.45 ^b^	30
Leucine	12.20 ± 2.51 ^a^	12.00 ± 1.72 ^a^	3.58 ± 0.82 ^b^	4.13 ± 0.93 ^b^	5.88 ± 0.76 ^b^	4.74 ± 0.37 ^b^	190
Tyrosine	30.04 ± 4.45 ^a^	19.86 ± 2.56 ^b^	5.59 ± 1.84 ^c^	8.88 ± 1.18 ^c^	10.23 ± 1.53 ^bc^	10.06 ± 1.32 ^bc^	91
Isoleucine	1.21 ± 0.62 ^c^	3.40 ± 0.03 ^a^	2.35 ± 0.98 ^b^	2.68 ± 0.50 ^b^	3.05 ± 0.91 ^a^	3.45 ± 1.22 ^a^	90
Phenylalanine	16.28 ± 2.37 ^a^	22.89 ± 1.28 ^a^	ND	ND	ND	ND	90
Lysine	22.36 ± 2.39 ^a^	3.17 ± 0.78 ^b^	4.72 ± 0.32 ^b^	4.37 ± 0.99 ^b^	5.89 ± 1.38 ^b^	4.44 ± 0.61 ^b^	50
Arginine	15.68 ± 1.14 ^a^	0.11 ± 0.03 ^b^	ND	ND	ND	ND	50
Histidine	13.89 ± 1.00 ^a^	10.30 ± 0.70 ^b^	ND	ND	ND	ND	20
∑BAA	116.61 ± 3.92 ^a^	92.15 ± 3.28 ^b^	19.42 ± 1.42 ^c^	22.53 ± 1.95 ^c^	28.45 ± 2.36 ^c^	26.57 ± 1.87 ^c^	/
Cystine	14.16 ± 1.91 ^b^	28.21 ± 2.86 ^a^	6.54 ± 0.88 ^c^	5.97 ± 0.68 ^c^	6.89 ± 1.38 ^c^	7.50 ± 1.40 ^c^	/
∑OAA	14.16 ± 1.91 ^b^	28.21 ± 2.86 ^a^	6.54 ± 0.88 ^c^	5.97 ± 0.68 ^c^	6.89 ± 1.38 ^c^	7.50 ± 1.40 ^c^	/
∑FAA	704.88 ± 28.72 ^b^	858.30 ± 30.56 ^a^	467.67 ± 15.83 ^c^	433.75 ± 16.18 ^c^	397.14 ± 13.05 ^cd^	361.02 ± 14.32 ^d^	/

Different lowercase letters in the same row indicate that there is significant difference (*p* < 0.05). ND: not detected; UAA: umami amino acid; SAA: sweet amino acid; BAA: bitter amino acid; OAA: other amino acid; FAA: free amino acid. “/”: not reported in the literature.

**Table 4:** Changes of 5′-nucleotides in lamb oyster cuts during Nang roasting (dry matter basis).

Nucleotide (mg/100 g)	Nang Roasting Stages						Taste Threshold (mg/100 g)
	Fresh Oyster Cuts	Marinated Oyster Cuts	Roasted-15	Roasted-30	Roasted-45	Roasted-60	
5′-GMP	0.39 ± 0.23 ^b^	0.82 ± 0.42 ^b^	2.82 ± 0.45 ^a^	1.90 ± 0.28 ^a^	2.35 ± 0.18 ^a^	2.30 ± 0.03 ^a^	12.50
5′-IMP	6.65 ± 2.01 ^b^	4.35 ± 1.80 ^b^	98.35 ± 37.39 ^a^	76.23 ± 22.25 ^a^	96.08 ± 21.17 ^a^	97.56 ± 3.39 ^a^	25
5′-AMP	5.38 ± 0.78 ^c^	4.69 ± 0.56 ^c^	23.64 ± 7.27 ^b^	31.97 ± 1.41 ^a^	34.34 ± 2.62 ^a^	30.85 ± 0.55 ^a^	50
5′-ADP	61.09 ± 3.35 ^b^	69.86 ± 7.11 ^a^	6.19 ± 1.79 ^c^	5.02 ± 0.64 ^c^	6.02 ± 1.21 ^c^	7.49 ± 0.99 ^c^	/
Hx	40.72 ± 7.81 b ^c^	196.38 ± 48.86 ^a^	69.16 ± 14.55 ^b^	23.55 ± 3.15 ^c^	22.17 ± 4.95 ^c^	15.77 ± 2.03 ^c^	/
I	18.47 ± 4.82 ^c^	17.79 ± 1.88 ^c^	45.87 ± 3.03 ^c^	80.4 ± 13.89 ^b^	124.45 ± 7.35 ^a b^	161.88 ± 31.08 ^a^	/
Flavor 5′-nucleotides	12.42 ± 3.79 ^b^	9.86 ± 1.93 ^b^	124.81 ± 28.04 ^a^	110.10 ± 1.10 ^a^	132.77 ± 18.10 ^a^	130.71 ± 2.96 ^a^	/
EUC (g MSG/100 g)	4.76 ± 0.23 ^b^	4.45 ± 0.51 ^b^	44.17 ± 5.28 ^a^	33.88 ± 2.41 ^a^	37.39 ± 4.13 ^a^	35.26 ± 2.89 ^a^	/

Different lowercase letters in the same row indicate that there is significant difference (*p* < 0.05). 5′-GMP: 5′-guanosine monophosphate, 5′-IMP: 5′-inosine monophosphate, 5′-AMP: 5′-adenosine monophosphate, 5′-ADP: 5′-adenosine diphosphate, Hx: hypoxanthine, I: inosine. The flavor 5′-nucleotides contained 5′-AMP, 5′-IMP and 5′-GMP.

## Data Availability

The data presented in this study are available on request from the corresponding author.

## References

[1] Roldan M., Ruiz J., del Pulgar J.S., Pérez-Palacios T., Antequera T. (2015). Volatile compound profile of sous-vide cooked lamb loins at different temperature–time combinations. Meat Sci..

[2] Xiao X., Hou C., Zhang D., Li X., Ren C., Ijaz M., Hussain Z., Liu D. (2020). Effect of pre- and post-rigor on texture, flavor, heterocyclic aromatic amines and sensory evaluation of roasted lamb. Meat Sci..

[3] Yue J., Zhang Y., Jin Y., Deng Y., Zhao Y. (2016). Impact of high hydrostatic pressure on non-volatile and volatile compounds of squid muscles. Food Chem..

[4] Petričević S., Radovčić N.M., Lukić K., Listeš E., Medić H. (2018). Differentiation of dry-cured hams from different processing methods by means of volatile compounds, physico-chemical and sensory analysis. Meat Sci..

[5] Han D., Mi S., Zhang C.-H., Li J., Song H.-L., Fauconnier M.-L., Tyteca E. (2019). Characterization and Discrimination of Chinese Marinated Pork Hocks by Volatile Compound Profiling Using Solid Phase Microextraction Gas Chromatography-Mass Spectrometry/Olfactometry, Electronic Nose and Chemometrics. Molecules.

[6] Liu Y., Zhang C., Chen S. (2013). Comparison of Active Non-volatile Taste Components in the Viscera and Adductor Muscles of Oyster (*Ostrea rivularis* Gould). Food Sci. Technol. Res..

[7] Liu H., Ma J., Pan T., Suleman R., Wang Z., Zhang D. (2021). Effects of roasting by charcoal, electric, microwave and superheated steam methods on (non)volatile compounds in oyster cuts of roasted lamb. Meat Sci..

[8] Song H., Liu J. (2018). GC-O-MS technique and its applications in food flavor analysis. Food Res. Int..

[9] Xu C.-H., Chen G.-S., Xiong Z.-H., Fan Y.-X., Wang X.-C., Liu Y. (2016). Applications of solid-phase microextraction in food analysis. TrAC Trends Anal. Chem..

[10] Xi J., Zhan P., Tian H., Wang P. (2019). Effect of Spices on the Formation of VOCs in Roasted Mutton Based on GC-MS and Principal Component Analysis. J. Food Qual..

[11] Wang Z., Cai R., Yang X., Gao Z., Yuan Y., Yue T. (2021). Changes in aroma components and potential Maillard reaction products during the stir-frying of pork slices. Food Control..

[12] Wall K.R., Kerth C.R., Miller R.K., Alvarado C. (2019). Grilling temperature effects on tenderness, juiciness, flavor and volatile aroma compounds of aged ribeye, strip loin, and top sirloin steaks. Meat Sci..

[13] Zhang L., Hu Y., Wang Y., Kong B., Chen Q. (2021). Evaluation of the flavour properties of cooked chicken drumsticks as affected by sugar smoking times using an electronic nose, electronic tongue, and HS-SPME/GC-MS. LWT.

[14] Zou Y., Kang D., Liu R., Qi J., Zhou G., Zhang W. (2018). Effects of ultrasonic assisted cooking on the chemical profiles of taste and flavor of spiced beef. Ultrason. Sonochem..

[15] Han D., Zhang C.-H., Fauconnier M.-L., Jia W., Wang J.-F., Hu F.-F., Xie D.-W. (2021). Characterization and comparison of flavor compounds in stewed pork with different processing methods. LWT.

[16] AOAC (2000). Official Methods of Analysis.

[17] Liu P., Wang S., Zhang H., Wang H., Kong B. (2019). Influence of glycated nitrosohaemoglobin prepared from porcine blood cell on physicochemical properties, microbial growth and flavour formation of Harbin dry sausages. Meat Sci..

[18] Liu H., Wang Z., Zhang D., Shen Q., Hui T., Ma J. (2020). Generation of key aroma compounds in Beijing roasted duck induced via Maillard reaction and lipid pyrolysis reaction. Food Res. Int..

[19] Liu Y., Xu X.-L., Zhou G.-H. (2007). Changes in taste compounds of duck during processing. Food Chem..

[20] Qi J., Liu D., Zhou G., Xu X. (2017). Characteristic Flavor of Traditional Soup Made by Stewing Chinese Yellow-Feather Chickens. J. Food Sci..

[21] Yamaguchi S., Yoshikawa T., Ikeda S., Ninomiya T. (1971). Measurement of the relative taste intensity of some l-?-amino acids and 5′-nucleotides. J. Food Sci..

[22] Yu T.-Y., Morton J., Clerens S., Dyer J. (2017). Cooking-Induced Protein Modifications in Meat. Compr. Rev. Food Sci. Food Saf..

[23] Trevisan A.J.B., Lima D.D.A., Sampaio G.R., Soares R.A.M., Bastos D.H.M. (2016). Influence of home cooking conditions on Maillard reaction products in beef. Food Chem..

[24] Khan M.I., Jo C., Tariq M.R. (2015). Meat flavor precursors and factors influencing flavor precursors—A systematic review. Meat Sci..

[25] Hu M., Wang S., Liu Q., Cao R., Xue Y. (2021). Flavor profile of dried shrimp at different processing stages. LWT.

[26] Watanabe A., Kamada G., Imanari M., Shiba N., Yonai M., Muramoto T. (2015). Effect of aging on volatile compounds in cooked beef. Meat Sci..

[27] Ying W., Ya-Ting J., Jin-Xuan C., Yin-Ji C., Yang-Ying S., Xiao-Qun Z., Dao-Dong P., Chang-Rong O., Ning G. (2016). Study on lipolysis-oxidation and volatile flavour compounds of dry-cured goose with different curing salt content during production. Food Chem..

[28] Ma Q., Hamid N., Oey I., Kantono K., Faridnia F., Yoo M., Farouk M. (2016). Effect of chilled and freezing pre-treatments prior to pulsed electric field processing on volatile profile and sensory attributes of cooked lamb meats. Innov. Food Sci. Emerg. Technol..

[29] Machiels D. (2004). Gas chromatography-olfactometry analysis of beef meat originating from differently fed Belgian Blue, Limousin and Aberdeen Angus bulls. Food Chem..

[30] Salum P., Guclu G., Selli S. (2017). Comparative Evaluation of Key Aroma-Active Compounds in Raw and Cooked Red Mullet (Mullus barbatus) by Aroma Extract Dilution Analysis. J. Agric. Food Chem..

[31] Benet I., Guàrdia M.D., Ibañez C., Solà J., Arnau J., Roura E. (2015). Analysis of SPME or SBSE extracted volatile compounds from cooked cured pork ham differing in intramuscular fat profiles. LWT.

[32] Li H., Li X., Zhang C.-H., Wang J.-Z., Tang C.-H., Chen L.-L. (2015). Flavor compounds and sensory profiles of a novel Chinese marinated chicken. J. Sci. Food Agric..

[33] Kawai M., Okiyama A., Ueda Y. (2002). Taste Enhancements between Various Amino Acids and IMP. Chem. Senses.

[34] Zheng J.-Y., Tao N.-P., Gong J., Gu S.-Q., Xu C.-H. (2015). Comparison of non-volatile taste-active compounds between the cooked meats of pre- and post-spawning Yangtze Coilia ectenes. Fish. Sci..

[35] Chen D.-W., Zhang M. (2007). Non-volatile taste active compounds in the meat of Chinese mitten crab (*Eriocheir sinensis*). Food Chem..

[36] Watanabe A., Tsuneishi E., Takimoto Y. (1989). Analysis of ATP and Its Breakdown Products in Beef by Reversed-Phase HPLC. J. Food Sci..

[37] Mateo J., Domínguez M.C., Aguirrezábal M.M., Zumalacárregui J. (1996). Taste compounds in chorizo and their changes during ripening. Meat Sci..

